# Reliability and physiological responsiveness of skin temperature and thermal contrast index during incremental exercise: a test–retest analysis

**DOI:** 10.1038/s41598-026-68741-x

**Published:** 2026-09-09

**Authors:** Lukas Masur, Hanna F. Willenbockel, Thekla Cordes, Billy Sperlich, Peter Düking

**Affiliations:** 1 Department of Sports Science and Movement Pedagogy, Technische Universität Braunschweig, Pockelsstrasse 11, 38102 Braunschweig, Germany; 2 Department of Bioinformatics and Biochemistry, Braunschweig Integrated Centre of Systems Biology (BRICS), Technische Universität Braunschweig, Braunschweig, Germany; 3 Research Group Cellular Metabolism in Infection, Helmholtz Centre for Infection Research, Braunschweig, Germany; 4 Integrative and Experimental Exercise Science & Training, Institute of Sport Science, University of Würzburg, Würzburg, Germany

**Keywords:** Cardiology, Health care, Medical research, Physiology

## Abstract

To investigate the test–retest reliability of skin temperature (T_sk_) and the thermal contrast index (TCI) during incremental exercise and examine their responsiveness to cardiovascular, metabolic, and perceptual parameters. Nineteen participants completed two standardized incremental cycling tests with concurrent thermographic, cardiovascular, metabolic, and perceptual measurements. T_sk_ and TCI reliability was evaluated using coefficients of variation, intraclass correlation coefficients, Bland–Altman analysis, and mixed-effects models. T_sk_ showed good to excellent reliability across all regions of interest (ICC_A,1_: 0.74–0.94 [CI: 0.44–0.98], SEM_A,1_: 0.28–0.53). TCI reliability varied by region and intensity, ranging from poor to good (ICC_A,1_: 0.23–0.80 [CI: -0.27-0.92], SEM_A,1_: 0.29–0.57), with a tendency toward higher reliability at greater exercise intensities and in the anterior thigh. Confidence intervals were generally wide, warranting cautious interpretation of the reliability estimates. TCI displayed consistent associations with heart rate (β = 0.46, *p* < 0.001), blood lactate (β = 0.17, *p* < 0.001), and energy expenditure (β = 0.14, *p* < 0.001). In contrast, T_sk_ was influenced more by individual traits than by physiological changes. TCI showed a tendency toward good reliability at higher intensities and in performance-relevant muscle regions, but lower reliability at low intensities and in less active areas. TCI also demonstrates meaningful responsiveness to internal load, particularly heart rate, highlighting its potential as a sensitive indicator of physiological load. In contrast, T_sk_ provides stable measurements but primarily reflects individual baseline characteristics rather than acute exercise responses.

## Introduction

Regular exercise is a key strategy to counteract noncommunicable diseases, reduce mortality, and enhance both health and physical performance^1^. However, metabolic inefficiency results in substantial heat production during exercise^2^. In the context of global warming, this poses increasing challenges, as elevated core temperature is associated with serious health risks, including heat exhaustion and heat stroke^3^. Understanding how the body dissipates heat is therefore as important as optimizing exercise performance, ensuring safety, and developing strategies to maintain physiological function under rising environmental temperatures. To investigate how the body dissipates heat from muscles to the skin, infrared thermography (IRT)^4^ can be used to quantify skin temperature (T_sk_) and visualize surface radiation patterns, namely the thermal contrast index (TCI), non-invasively, rapidly and at low-cost^5^, thereby allowing insights into thermoregulatory responses on large scale.

During high-intensity endurance exercise, T_sk_ typically declines, reflecting peripheral vasoconstriction of arterioles that redirects blood flow toward the active musculature to meet increasing oxygen demands^6,7^. In contrast, the development of TCI^5^ is associated with the activation of individual perforator vessels through cholinergic nerve transmission and the non-adrenergic vasodilator system, resulting in the emergence of vascular territories (“perforasomes”) that promote efficient heat dissipation^8^. Although the kinetics of T_sk_ during exercise have been researched^9–11^, only recent research reported a steady increase in TCI during an incremental cycling test and a decline in the 15 min recovery period^5^, reinforcing previous established theories^8^. Expanding on previous studies that examined associations between T_sk_ and physiological parameters such as lactate, heart rate, maximal oxygen uptake (V̇O_2max_), and rating of perceived exertion (RPE)^9,12,13^, recent evidence indicates that TCI correlates more strongly with markers of internal load, including heart rate and oxygen uptake than T_sk_^5^. This suggests that TCI may represent a more sensitive indicator of internal load and is more tightly regulated than T_sk_.

While this research has increased the understanding of the development of TCI in conjunction with T_sk_ and their relationship with other internal load parameters during exercise, it is currently unknown if results can be replicated reliably. However, such research is crucial to determine if kinetics of T_sk_ and TCI assessed by IRT, as well as their relationship to other internal load markers, are consistent under the same conditions and exercise. Only if results are reliable, deviations of TCI and T_sk_ assessed by IRT could be useful to inform decision making in sports practice, e.g., to determine if current exercise exposes the individual to increased thermoregulatory load or if a training mesocycle improved the appearance of TCI and consequently the ability of an individual to dissipate heat.

Consequently, the aims of the study were:

i. to investigate the reliability of TCI and T_sk_ during and following two standardized incremental exercise tests.
ii. to elucidate whether selected cardiovascular, metabolic, and subjective parameters affect TCI and T_sk_ similarly between repeated tests.

Based on the aforementioned information, we hypothesized that both TCI and T_sk_ assessed by infrared thermography would demonstrate acceptable test–retest reliability across two identical incremental cycling tests, with T_sk_ showing higher relative and absolute reliability than TCI. Furthermore, we hypothesized that TCI would exhibit stronger and more consistent associations with cardiovascular and metabolic markers of internal load (e.g., heart rate, lactate, and V̇O₂) than T_sk_, indicating its greater sensitivity to acute physiological responses during exercise.

## Methods

### Participants

For this study, a total of 19 trained male participants were enrolled. In accordance with the Declaration of Helsinki, each participant received a detailed verbal and written information about the study procedures and gave written informed consent. The study was approved by the local ethics committee for research involving human subjects (approval number: D-2024-07). In accordance with the Thermographic Imaging in Sports and Exercise Medicine (TISEM) checklist^14^ and the recommendations of Fernández-Cuevas et al.^15^, participants were required to abstain from alcohol, smoking, caffeine, large meals within 1 h before testing, as well as from ointments, cosmetics, and showering within 4 h. Physiotherapeutic or physical treatments (e.g., massage, heat or cold therapy, electrostimulation), sun exposure, and strenuous exercise were prohibited for at least 24 h prior to testing. Participants presenting with any signs of illness or injury were excluded.

### Procedures

#### Experimental design and exercise testing

**Fig. 1:**
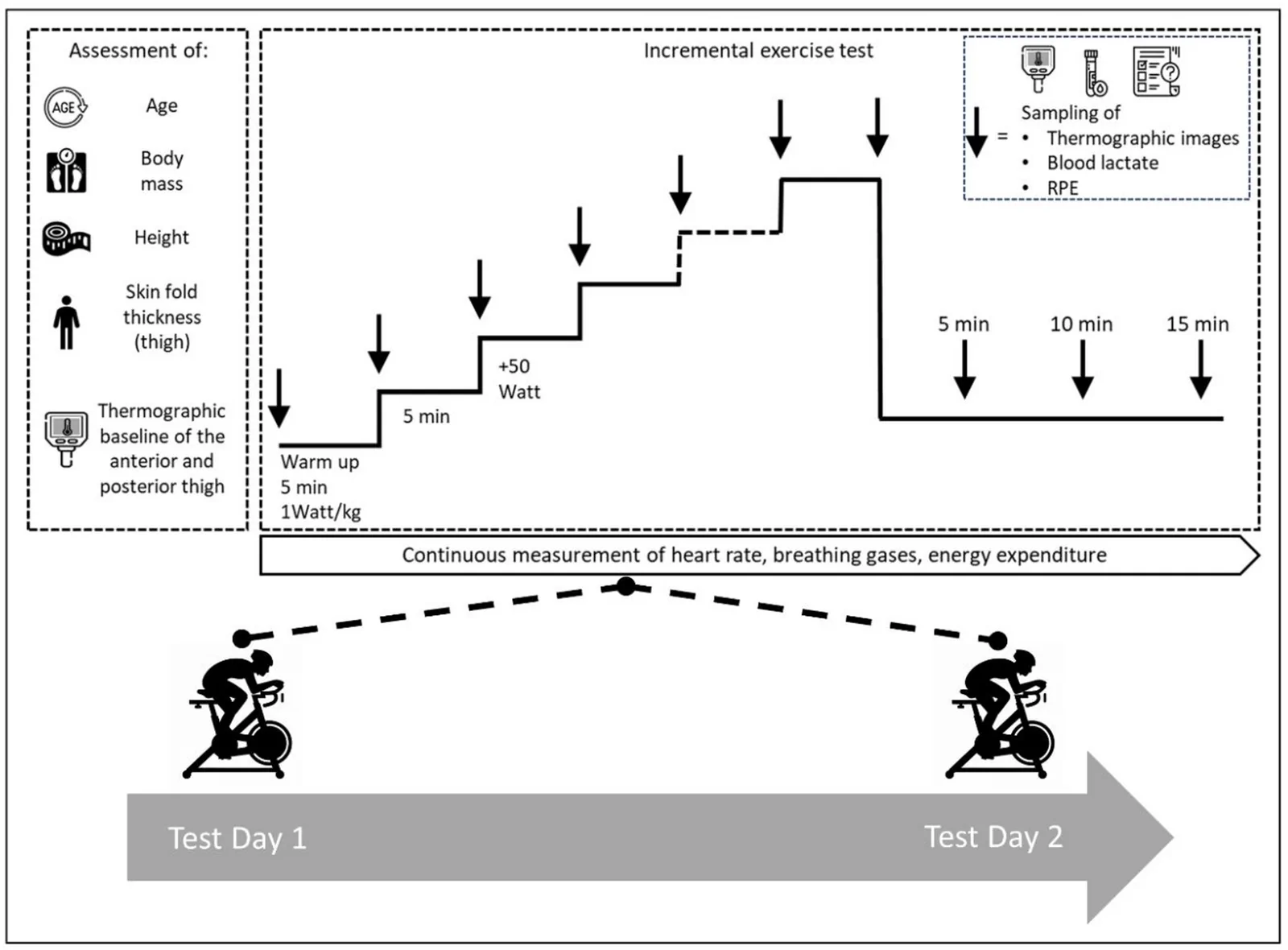
Schematic representation of experimental procedures (adapted from Masur et al.^5^). *RPE* rating of perceived exertion.

Experimental procedures are schematically illustrated in Fig. 1. This investigation was part of a larger research project, examining the response of IRT-related parameters during cycling^5^.

This test-retest study encompassed two incremental exercise tests performed on a stationary cycle ergometer (Cyclus 2, RBM, Leipzig, Germany), equipped with an electromagnetic braking system and a power measurement accuracy of ± 2%, as specified by the manufacturer.

#### Incremental exercise test protocol

The incremental exercise test began with a 5-min warm-up at 1 W·kg⁻¹, after which the test started at 100 W with 50 W increases every 5 min until volitional exhaustion. Participants remained seated and maintained a pedal cadence of 80 rpm. Maximal exertion and V̇O_2max_ attainment were assumed if at least three of the following criteria were met: (i) an oxygen consumption (V̇O_2_) plateau despite increased workload and ventilation, (ii) blood lactate ≥ 9 mmol·L⁻¹, (iii) respiratory exchange ratio ≥ 1.10, and (iv) heart rate > 95% of the age-predicted maximum.

#### Anthropometrics and physiological analyses

Before the incremental exercise test, anthropometric measurements were taken, including body weight, height, and thigh skinfold thickness. Since our study exclusively assessed thermographic parameters of the thigh region, inter-individual variability of the thigh skinfold thickness has been analyzed. Regarding the impact of overall body fat percentage on thermographic images, we refer to previous research^15,16^. Body weight was assessed using a bioelectrical impedance device (Tanita BC-601, Tokyo, Japan), height with a standard measuring tape, and thigh skinfold thickness via calipers following established anthropometric protocols. Pulmonary gas exchange, including V̇O₂ and carbon dioxide production (V̇CO₂), was measured breath-by-breath using a calibrated gas analyzer (Cortex Metamax 3B R2, Cortex Biophysik, Leipzig, Germany). Calibration was performed before each test with a certified gas mixture (15.8% O₂, 5% CO₂ in N₂; Praxair, Düsseldorf, Germany) and a 3-L syringe was used to verify flow volume accuracy. According to the manufacturer, the oxygen sensor of the analyzer operates with a technical measurement error below 3%^17,18^. Heart rate was continuously monitored using the gas analyzer’s integrated telemetry system with a validated chest strap (Polar H10, Polar Electro Oy, Kempele, Finland), and all calibrations followed manufacturer specifications. Capillary blood from the right earlobe was collected for lactate analysis (Lactate Pro 2, Arkray KDK, Kyoto, Japan) prior to testing, after each 5-min stage, immediately post-exercise, and at 5, 10, and 15 min of recovery. At the same time points, participants recorded their perceived exertion using the Borg scale (6–20)^19^.

#### Thermographic measurement and analysis

The participants were wearing light sportswear not compressing adjacent tissue. For thermal image measurements and as used in other scientific articles^20–23^, the uncooled FLIR E54 IR camera (Wilsonville, OR, USA) with noise-equivalent temperature difference < 40 mK, 320 × 240 pixels of resolution was used. According to the manufacturer, the temperature accuracy of the device is ± 2% or ± 2 °C. Standardized thermal image recording was accomplished by the adherence of the TISEM checklist^14^ and recommendations of Fernandez Cuevas^15^. The camera was turned on at least 30 min prior every measurement and placed perpendicular to the region of interest (ROI) using a tripod at a distance of 2.80 m from the participant. Average temperature and humidity were recorded on each testing day. The participants acclimatized to room temperature and humidity for approximately 20 min prior to the test in a standing position. Thermal images of the anterior and posterior lower limb were taken pre, after each 5-min step, immediately after the test and 5, 10 and 15 min into the recovery period. Thermographic analysis was performed with the approved software (Thermacam Researcher Pro 2.10 software, FLIR, Wilsonville, Oregon, USA), yielding the mean and standard deviation of T_sk_. The ROIs were defined based on anatomical criteria established in previous studies^24,25^. The anterior and posterior thigh were delineated by drawing a straight line from the groin area towards the lateral side of the thigh, selecting the largest possible area bounded superiorly by the upper border of the patella^25^. TCI was calculated according to an equation previously applied in the literature^8^:

$$\:TCI=10\%\:darkest\:pixels-10\%\:lightest\:pixels\:within\:the\:region\:of\:interest$$

### Statistical analysis

Normality and homoscedasticity were assessed for both TCI and T_sk_ prior to reliability and linear mixed model analyses. Shapiro-Wilk tests on the differences between Test 1 and Test 2 indicated that TCI differences were slightly non-normal (W = 0.995, *p* = 0.032), whereas T_sk_ differences showed stronger deviation from normality (W = 0.982, *p* < 0.001). Levene’s test for homogeneity of variance confirmed equal variances between Test 1 and Test 2 for TCI (F = 0.017, *p* = 0.896) and showed no significant deviation from homoscedasticity for T_sk_ (F = 3.213, *p* = 0.073). These results suggest minor departures from normality; however, given the robustness of linear mixed models and reliability metrics (ICC, CV, SEM, MDC) to moderate non-normality, the analyses were considered valid. Although the Shapiro–Wilk test indicated a statistically significant deviation from normality for the T_sk_ differences (W = 0.982, *p* < 0.001), visual inspection of histograms and Q–Q plots suggested only minor departures from normality. Given the large number of observations, where normality tests are highly sensitive to small deviations, the conventional Bland–Altman limits of agreement were retained. No a priori sample size calculation was performed, as no previous reliability data were available for TCI and only limited methodological evidence existed for T_sk_^26^. The sample size was determined by the number of eligible participants available during the study period.

Proportional bias was assessed using a linear mixed-effects model of Test 1–Test 2 differences against the mean of both measurements, including subject as a random intercept. A small but statistically significant mean-dependent bias was observed for TCI (β = 0.061, *p* = 0.001), indicating that measurement differences increased slightly at higher TCI values. The analysis for T_sk_ also demonstrated a small, but statistically significant mean-dependent bias (β = 0.106 °C per °C increase in mean T_sk_, *p* < 0.001). However, the magnitude of this effect was small relative to the overall limits of agreement and measurement error.

To investigate the reliability of TCI and T_sk_, several complementary statistical metrics were used: (i) the coefficient of variation (CV) to assess absolute reliability, (ii) the intraclass correlation coefficient (ICC) to assess relative reliability, (iii) the standard error of measurement (SEM), (iv) the minimal detectable change at the 95% confidence level (MDC95), and (v) Bland–Altman analysis to evaluate agreement between repeated measurements. As previously demonstrated^26^, absolute-agreement ICCs (ICC_A_) were calculated using a two-way mixed-effects model following the framework of McGraw and Wong^27^ for both single measurements (ICC_A,1_) and the average of two measurements (ICC_A,2_). ICC_A,1_ reflects the reliability of a single thermographic measurement, whereas ICC_A,2_ reflects the reliability of the average of two repeated measurements. Because the aim was to evaluate agreement between repeated measurements rather than consistency alone, the absolute-agreement ICC model was considered the most appropriate. ICC values were interpreted as follows: <0.50 poor, 0.50–0.75 moderate, 0.75–0.90 good, and 0.90–1.00 excellent^28^.

The SEM_A1_, SEM_A2_^28^, MDC95_A1_, and MDC_A2_^29^ were calculated using the ICC_A,1_ and ICC_A,2_ values:

$$\:SEM=SD\times\:\sqrt{1-{ICC}_{A}}$$

$$\:MDC95=1.96\times\:\sqrt{2}\times\:SEM\:=2.77\times\:SEM$$

A linear mixed-effects model was used to evaluate the consistency of the effects of selected cardiovascular, metabolic, and subjective parameters on TCI and T_sk_.

The CV ^30^ was calculated as:

$$\:CV\:\left(\%\right)=\frac{SD}{Mean\:}\times\:100.$$

Bland–Altman plots were used to visualize agreement between repeated measurements, including mean bias and 95% limits of agreement. A linear mixed-effects model (LMM)^31^ was used to examine the effects of selected cardiovascular, metabolic, and subjective parameters (lactate, V̇O_2_, heart rate, energy expenditure, and RPE) across the summarized test increments (Pre, Warm-up, 100–200 W, 250–350 W, Post 5–15 min) and their interaction effects between the two testing occasions on TCI and T_sk_. The models included random intercepts for Subject to account for repeated measures across the two test occasions. Fixed effects included all interaction terms between test day and each cardiovascular, metabolic or subjective parameter to evaluate whether these associations differed between testing occasions. Visual inspection of Q–Q plots and histograms of the residuals suggested approximate normality^32,33^. To directly compare the relative influence of selected cardiovascular, metabolic and subjective parameters on T_sk_ and TCI, we standardized all variables by calculating z-scores. This converts each variable to a common scale (mean = 0, SD = 1), allowing the regression coefficients to reflect effect sizes rather than units. For the analysis of relative predictor influence on TCI and T_sk_, variables were standardized using z-scores. The test-retest comparison models were performed using the original measurement units to allow interpretation of between-day changes in physiologically meaningful units. The analysis was performed using Rstudio (version 2024.12.0, Posit Software, Boston, MA, USA).

## Results

In the study a total of 19 participants performed two incremental cycling tests and showed the following anthropometric measures (all mean ± standard deviation): age; 28.1 ± 8.6 years, height; 182.6 ± 6.9 cm, body mass; 76.5 ± 7.4 kg, body mass index; 23.0 ± 1.8 kg m^−2^, skinfold thickness thigh; 9.5 ± 3.5 mm. The observed variability in thigh skinfold thickness was considered acceptable and therefore not further investigated. Mean V̇O_2max_ and mean maximal heart rate were 58.9 ± 7.4 ml min^−1^ kg^−1^ and 184.0 ± 10.9 bpm, respectively. The mean ambient temperature was 23.5 ± 1.3 °C and the mean humidity was 52.5 ± 0.1% during the two incremental cycling tests.

**Fig. 2:**
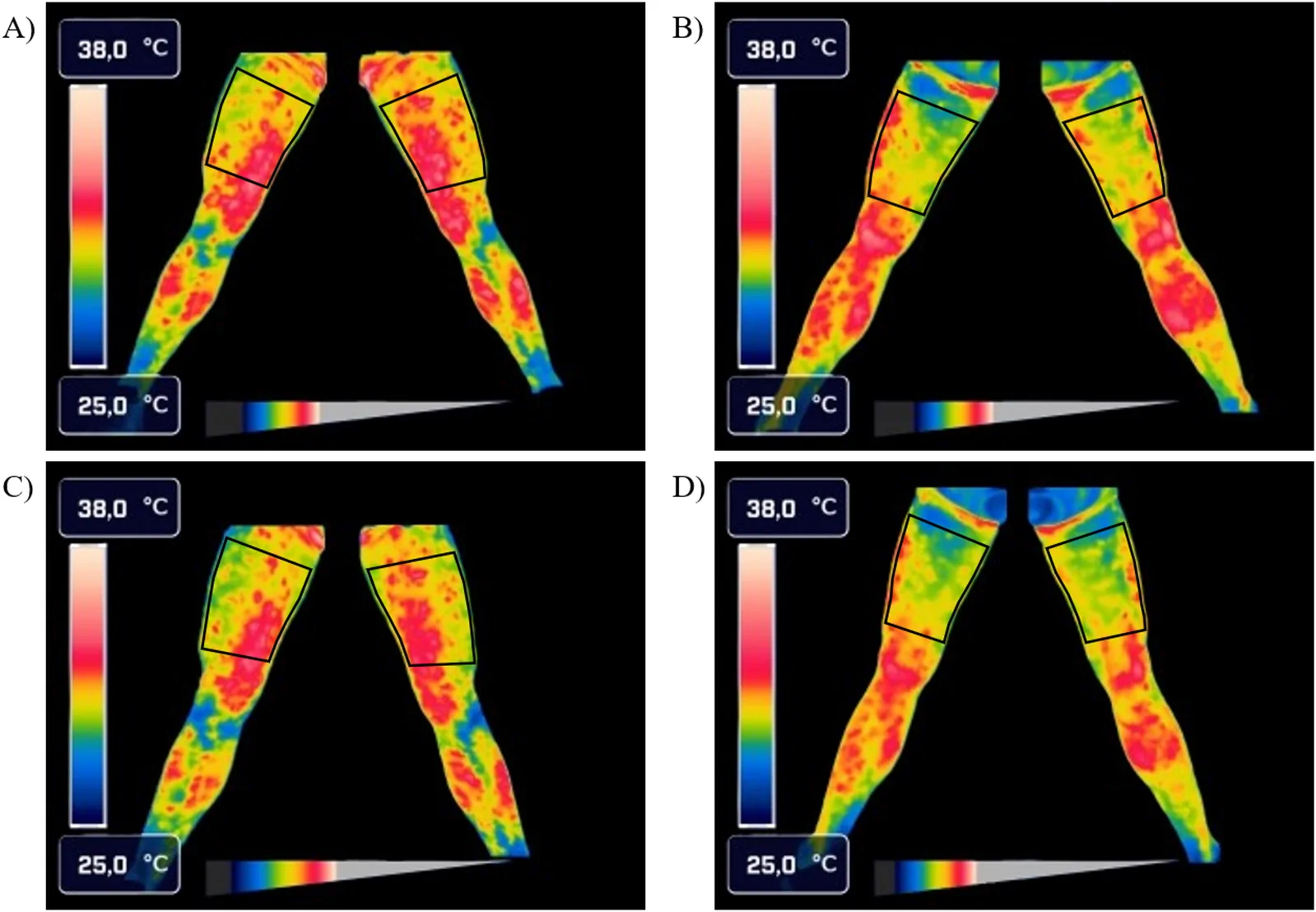
Representative thermographic images of the anterior (**A**, **C**) and posterior (**B**, **D**) views of one athlete’s lower extremities at the 200 W increment of Test 1 (**A**, **B**) and Test 2 (**C**, **D**). Selected ROIs are depicted in each image.

Figure 2 depicts representative thermographic images from a single participant at the 200 W increment of Test 1 (Fig. 2A,B) and Test 2 (Fig. 2C,D).

**Fig. 3:**
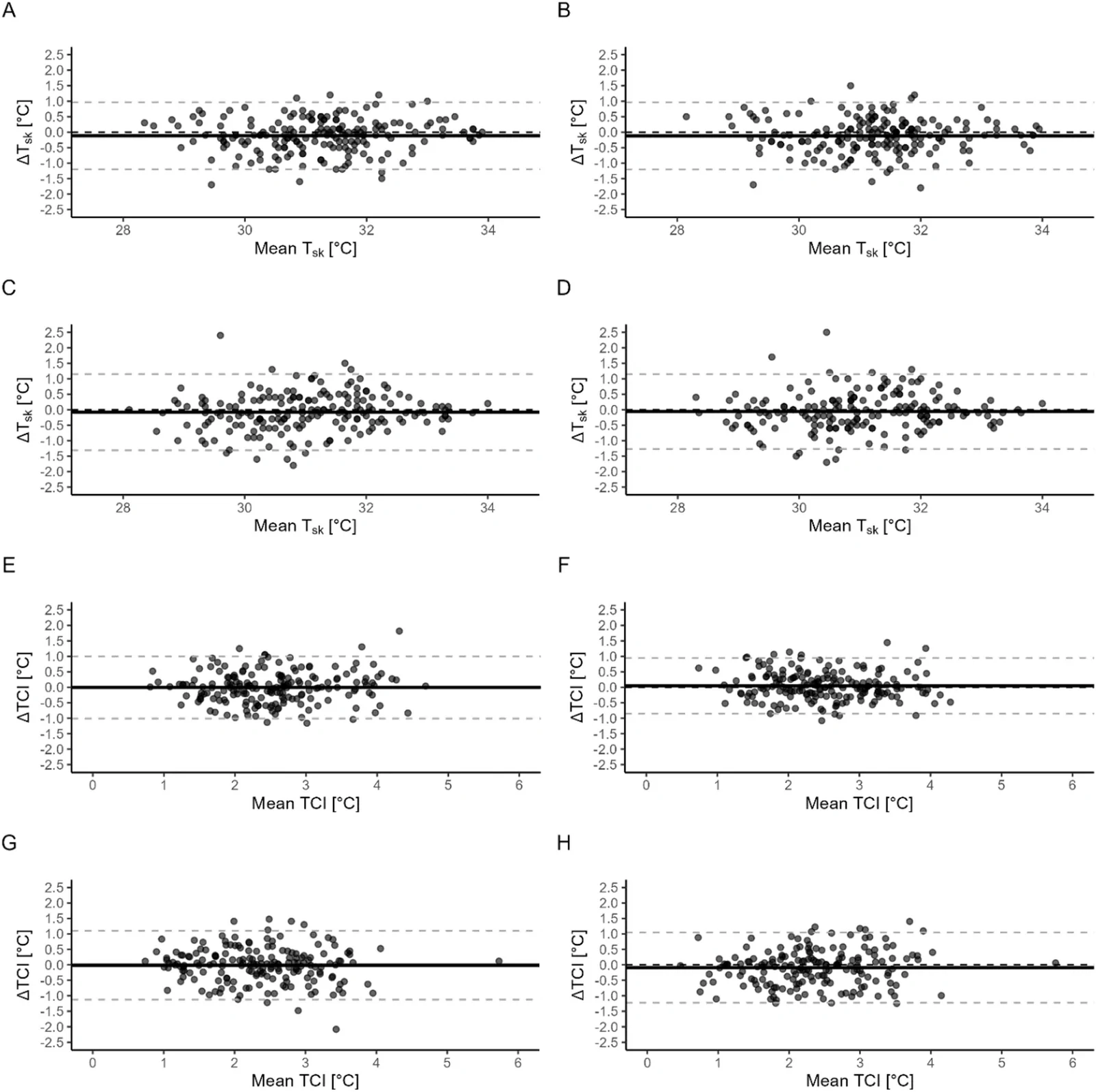
Bland–Altman plots depicting the test–retest agreement for T_sk_ (**A**–**D**) and TCI (**E**–**H**) of all test increments (*n* = 19). (**A**,**E**) Right anterior thigh. (**B**,**F**) Left anterior thigh. (**C**, **G**) Right posterior thigh. (**D**, **H**) Left posterior thigh. Bold line indicates the mean difference (bias) between the two measurements. Dashed lines indicate the limits of agreement (mean ± 1.96 × SD). X-axis starts at the minimum physiologically meaningful value. TCI, Thermal contrast index; T_sk_, Skin temperature.

Bland-Altman plots for TCI and T_sk_ (Fig. 3) illustrate the agreement between measurements obtained during and following the two incremental exercise tests, showing the mean differences (bias) and 95% limits of agreement for each parameter.

Our results showed good to excellent relative reliability of T_sk_ across all ROIs and exercise increments, with ICC_A,1_ values between 0.74 and 0.94 (CI: 0.44–0.98). The highest relative consistency was observed during 100–350 W (ICC_A,1_: 0.86–0.94 [CI: 0.54–0.98]), exceeding Pre values (ICC_A,1_: 0.74–0.77 [CI-0.44-0.90]) (Table 2). For TCI, reliability was higher in the anterior thigh (100–350 W: ICC_A,1_: 0.62–0.80 [CI: 0.09–0.92]) compared to the posterior thigh (100–350 W: ICC_A,1_: 0.54–0.57 [CI: -0.06-0.87]). Anterior thigh reliability during 100–350 W (ICC_A,1_: 0.62–0.80 [CI: 0.09–0.92) exceeded Pre (ICC_A,1_: 0.23–0.67 [CI: -0.27-0.86]), Warm-up (ICC_A,1_: 0.44–0.59 [CI: 0.01–0.82]), and Post 5–15 min (ICC_A,1_: 0.36–0.66 [CI: -0.13-0.86]) (Table 1).

**Table 1:** Results of the TCI repeatability analysis.

ROI	Increment	ICC_A,1_ [CI]	ICC_A,2_ [CI]	CV	SEM_A,1_	MDC95_A,1_	SEM_A,2_	MDC95_A,2_
Right anterior thigh	Pre	0.67 [0.31–0.86]	0.80 [0.48–0.92]	14.90	0.31	0.86	0.24	0.67
Right anterior thigh	Warm-up	0.59 [0.19–0.82]	0.74 [0.32–0.90]	14.91	0.32	0.88	0.25	0.70
Right anterior thigh	100–200 W	0.80 [0.55–0.92]	0.89 [0.70–0.96]	9.87	0.32	0.88	0.24	0.66
Right anterior thigh	250–350 W	0.74 [0.18–0.92]	0.85 [0.24–0.96]	8.48	0.49	1.37	0.38	1.05
Right anterior thigh	Post 5–15 min	0.63 [0.25–0.84]	0.76 [0.36–0.91]	14.03	0.48	1.32	0.38	1.05
Left anterior thigh	Pre	0.46 [0.01–0.75]	0.63 [0.01–0.86]	23.24	0.39	1.09	0.33	0.90
Left anterior thigh	Warm-up	0.55 [0.16–0.80]	0.71 [0.26–0.89]	12.84	0.29	0.81	0.24	0.65
Left anterior thigh	100–200 W	0.76 [0.50–0.90]	0.86 [0.64–0.95]	8.42	0.30	0.82	0.23	0.62
Left anterior thigh	250–350 W	0.62 [0.09–0.89]	0.75 [-0.06-0.94]	8.91	0.57	1.57	0.46	1.27
Left anterior thigh	Post 5–15 min	0.66 [0.30–0.86]	0.79 [0.40–0.92]	12.48	0.41	1.14	0.32	0.90
Right posterior thigh	Pre	0.23 [-0.27-0.61]	0.37 [-0.73-0.76]	20.55	0.36	1.00	0.33	0.93
Right posterior thigh	Warm-up	0.45 [0.01–0.74]	0.62 [0.02–0.85]	16.42	0.36	1.00	0.30	0.83
Right posterior thigh	100–200 W	0.55 [0.13–0.80]	0.70 [0.21–0.89]	12.74	0.42	1.15	0.34	0.94
Right posterior thigh	250–350 W	0.57 [-0.06-0.87]	0.72 [-0.19-0.93]	8.65	0.55	1.53	0.45	1.24
Right posterior thigh	Post 5–15 min	0.36 [-0.13-0.70]	0.52 [-0.31-0.83]	15.16	0.55	1.52	0.47	1.31
Left posterior thigh	Pre	0.35 [-0.06-0.68]	0.52 [-0.15-0.81]	30.67	0.43	1.19	0.37	1.02
Left posterior thigh	Warm-up	0.44 [0.01–0.74]	0.61 [0.03–0.85]	16.93	0.32	0.90	0.27	0.75
Left posterior thigh	100–200 W	0.54 [0.14–0.80]	0.68 [0.14–0.89]	12.26	0.45	1.26	0.38	1.04
Left posterior thigh	250–350 W	0.54 [-0.06-0.86]	0.69 [-0.53-0.93]	12.17	0.57	1.59	0.47	1.30
Left posterior thigh	Post 5–15 min	0.43 [-0.02-0.74]	0.59 [-0.11-0.85]	15.70	0.52	1.44	0.44	1.22

*CI* Confidence interval; *CV* Coefficient of variation; *ICC* Intraclass correlation coefficient; *MDC95* Minimal detectable change at 95% confidence; *ROI*, Region of interest; *SEM* Standard error of measurement; *W* Watt.

**Table 2:** Results of the T_sk_ repeatability analysis.

ROI	Increment	ICC_A,1_ [CI]	ICC_A,2_ [CI]	CV	SEM_A,1_	MDC95_A,1_	SEM_A,2_	MDC95_A,2_
Right anterior thigh	Pre	0.76 [0.48–0.90]	0.87 [0.65–0.95]	1.19	0.46	1.26	0.34	0.95
Right anterior thigh	Warm-up	0.85 [0.65–0.94]	0.92 [0.79–0.97]	1.02	0.36	1.01	0.27	0.74
Right anterior thigh	100–200 W	0.92 [0.80–0.97]	0.96 [0.89–0.98]	0.71	0.29	0.79	0.21	0.57
Right anterior thigh	250–350 W	0.94 [0.78–0.98]	0.97 [0.87–0.99]	0.83	0.34	0.94	0.24	0.67
Right anterior thigh	Post 5–15 min	0.85 [0.60–0.95]	0.92 [0.73–0.97]	1.31	0.48	1.34	0.36	0.99
Left anterior thigh	Pre	0.74 [0.44–0.89]	0.85 [0.61–0.94]	1.24	0.47	1.30	0.36	0.99
Left anterior thigh	Warm-up	0.90 [0.75–0.96]	0.95 [0.86–0.98]	0.78	0.30	0.84	0.22	0.61
Left anterior thigh	100–200 W	0.92 [0.82–0.97]	0.96 [0.90–0.98]	0.70	0.28	0.78	0.20	0.56
Left anterior thigh	250–350 W	0.91 [0.67–0.98]	0.96 [0.80–0.99]	1.01	0.38	1.05	0.27	0.76
Left anterior thigh	Post 5–15 min	0.85 [0.60–0.94]	0.92 [0.73–0.97]	1.29	0.48	1.33	0.36	0.99
Right posterior thigh	Pre	0.77 [0.49–0.90]	0.87 [0.65–0.95]	1.00	0.41	1.13	0.31	0.85
Right posterior thigh	Warm-up	0.86 [0.68–0.94]	0.93 [0.81–0.97]	0.89	0.36	1.00	0.26	0.73
Right posterior thigh	100–200 W	0.90 [0.78–0.96]	0.95 [0.87–0.98]	0.87	0.33	0.91	0.24	0.66
Right posterior thigh	250–350 W	0.86 [0.54–0.97]	0.92 [0.64–0.98]	1.26	0.50	1.40	0.38	1.04
Right posterior thigh	Post 5–15 min	0.80 [0.47–0.93]	0.89 [0.59–0.96]	1.48	0.53	1.47	0.40	1.10
Left posterior thigh	Pre	0.75 [0.46–0.90]	0.86 [0.63–0.95]	1.03	0.40	1.11	0.30	0.83
Left posterior thigh	Warm-up	0.88 [0.71–0.95]	0.93 [0.83–0.97]	0.85	0.34	0.93	0.25	0.68
Left posterior thigh	100–200 W	0.86 [0.68–0.94]	0.92 [0.78–0.97]	0.90	0.40	1.12	0.31	0.85
Left posterior thigh	250–350 W	0.90 [0.65–0.97]	0.94 [0.78–0.99]	1.14	0.42	1.17	0.31	0.85
Left posterior thigh	Post 5–15 min	0.81 [0.45–0.94]	0.90 [0.57–0.97]	1.34	0.52	1.43	0.39	1.07

*CI* Confidence interval; *CV* Coefficient of variation; *ICC* Intraclass correlation coefficient; *MDC95* Minimal detectable change at 95% confidence; *ROI*, Region of interest; *SEM* Standard error of measurement; *W* Watt.

The linear mixed model results (Table 3) indicated that higher lactate (β = 0.17, 95% CI [0.10, 0.24], *p* < 0.001) and heart rate (β = 0.46, 95% CI [0.33, 0.60], *p* < 0.001) were positively associated with standardized TCI, while V̇O₂ showed no significant effect (*p* = 0.884). For T_sk_, lactate (β = -0.12, 95% CI [-0.18, -0.06], *p* < 0.001), V̇O_2_ (β = -0.20, *p* < 0.001), and RPE (β = -0.30, *p* < 0.001) were negatively associated, while heart rate was positively associated (β = 0.24, *p* < 0.001). Significant interactions indicated that the effect of lactate and heart rate on T_sk_ differed between test days (*p* = 0.010 and *p* = 0.002, respectively). The outcomes on TCI demonstrated that the residual variance (σ² = 0.48) exceeded the random intercept variance (τ₀₀ = 0.17), resulting in an ICC of 0.27, indicating relatively low between-subject variance. In contrast, the linear mixed model on T_sk_ showed a much larger random intercept variance (τ₀₀ = 0.67) compared to residual variance (σ² = 0.28), corresponding to an ICC of 0.70 and indicating strong subject-specific baselines. The fixed effects explained 41.2% of variance in TCI and 10.4% in T_sk_ (marginal R²), while including random effects increased the explained variance to 56.8% and 73.5%, respectively (conditional R²) (Table 3).

**Table 3:** Results of the linear mixed-effects model examining the impact of physiological parameters and their interactions between test condition on TCI and T_sk_.

Predictors	TCI (standardized)					T_sk_ (standardized)				
	Estimates	Std. Error	CI	t value	*p* value	Estimates	Std. Error	CI	t value	*p* value
Intercept	-0.07	0.10	-0.26–0.13	-0.69	0.493	-0.00	0.19	-0.37–0.37	-0.00	0.996
Test Day 2	0.07	0.04	-0.00–0.15	1.88	0.060	0.10	0.03	0.04–0.16	3.27	**0.001**
Lactate	0.17	0.04	0.10–0.24	4.63	**< 0.001**	-0.11	0.03	-0.18 – -0.05	-3.68	**< 0.001**
V̇O_2_	-0.01	0.06	-0.13–0.11	-0.15	0.884	-0.21	0.05	-0.32 – -0.11	-3.93	**< 0.001**
Heart rate	0.46	0.07	0.33–0.60	6.64	**< 0.001**	0.26	0.06	0.14–0.37	4.36	**< 0.001**
Energy expenditure	0.14	0.03	0.08–0.21	4.41	**< 0.001**	-0.08	0.03	-0.13 – -0.02	-2.82	**0.005**
RPE	0.11	0.07	-0.02–0.25	1.64	0.101	-0.29	0.06	-0.41 – -0.18	-5.04	**< 0.001**
Test2:Lactate	-0.01	0.05	-0.11–0.09	-0.22	0.826	0.11	0.04	0.03–0.19	2.58	**0.010**
Test2:V̇O_2_	0.01	0.08	-0.14–0.17	0.13	0.896	0.02	0.07	-0.11–0.15	0.32	0.751
Test2:Heart rate	0.10	0.08	-0.07–0.26	1.16	0.246	-0.22	0.07	-0.35 – -0.09	-3.23	**0.001**
Test2:Energy expenditure	0.02	0.04	-0.07–0.10	0.41	0.685	0.04	0.04	-0.03–0.11	1.08	0.281
Test2:RPE	-0.14	0.09	-0.32–0.04	-1.55	0.122	0.13	0.08	-0.02–0.28	1.74	0.081
Random Effects										
σ^2^	0.48					0.28				
τ_00_	0.17 _Subject_					0.67 _Subject_				
Marginal R^2^ / Conditional R^2^	0.412 / 0.568					0.104 / 0.735				

*CI*, Confident interval; *RPE*, Rating of perceived exertion; *TCI*, Thermal contrast index; *T*_*sk*_, Skin temperature; *VO*_2_, Oxygen consumption.

**Table 4:** Linear mixed model results of the effect of test condition on physiological parameters.

Effect	Estimate	Std. error	Df	t value	*p* value
Lactate	-0.12	0.17	1343.88	-0.73	0.47
V̇O_2_	-0.38	0.84	1343.66	-0.45	0.65
Heart rate	-3.53	1.55	1343.77	-2.27	**0.02**
Energy expenditure	-11.69	18.69	1343.42	-0.63	0.53
RPE	-0.02	0.22	1344.08	-0.07	0.94

*Df* degrees of freedom; *RPE* Rating of perceived exertion; *VO*_*2*_ Oxygen consumption.

A linear mixed-effects model revealed a significant difference in heart rate between the two test conditions (Test 1 vs. Test 2), with heart rate being on average 3.53 bpm lower in Test 2 (Estimate = -3.53, SE = 1.55, *t*(1344) = − 2.27, *p* = 0.023). A random intercept for subject was included to account for repeated measures (*SD* = 10.21) (Table 4).

## Discussion

The aims of the study were (i) to investigate the reliability of TCI and T_sk_ during and following two standardized incremental exercise tests and (ii) to elucidate whether selected cardiovascular, metabolic, and subjective parameters affect TCI and T_sk_ similarly between repeated tests. The main results of the study were:

1. Over the different exercise intensities, T_sk_ showed better reliability (ICC_A,1_: 0.74–0.94 [CI: 0.44–0.98], SEM_A,1_: 0.28–0.53) compared to all TCI ROIs (ICC_A,1_: 0.23–0.80 [CI: -0.27-0.92], SEM_A,1_: 0.29–0.57). For TCI, reliability showed a tendency to increase with increasing exercise intensity (ICC_A,1_ up to 0.80, depending on ROI), and tends to be more reliable in anterior (ICC_A,1_: up to 0.80) compared to posterior thigh (ICC_A,1_: up to 0.57). Confidence intervals were generally wide, warranting cautious interpretation of the reliability estimates (see Tables 1 and 2).
2. TCI was significantly and consistently influenced by lactate (β = 0.17, *p* < 0.001), heart rate (β = 0.46, *p* < 0.001) and energy expenditure (β = 0.14, *p* < 0.001). T_sk_ was significantly influenced by lactate (β = -0.11, *p* < 0.001), heart rate (β = 0.26, *p* < 0.001), V̇O_2_ (β = -0.21, *p* < 0.001), energy expenditure (β = -0.08, *p* = 0.005) and RPE (β = -0.29, *p* < 0.001). Exploratory interaction analyses indicated that the associations of T_sk_ with lactate (β = 0.11, *p* = 0.010) and heart rate (β = -0.22, *p* = 0.001) differed between testing conditions, suggesting an inconsistent responsiveness of T_sk_ to lactate and heart rate between the two test occasions.
3. TCI is more responsive to physiological changes (explained variation: σ² = 0.48 > τ₀₀ = 0.17; marginal and conditional R^2^: 0.412, 0.568) compared to T_sk_ which is more influenced by subject-specific characteristics (explained variation: τ₀₀ = 0.67 > σ² = 0.28; marginal and conditional R^2^: 0.104, 0.735), suggesting that TCI better reflects acute physiological fluctuations across repeated tests, while T_sk_ represents a stable individual baseline.

### Reliability of TCI and its responsiveness to physiological parameters following two incremental cycling tests

How the body dissipates heat is becoming increasingly important. While it is acknowledged that heat needs to be dissipated by the skin during intense exercise, decreasing skin temperature indicates that blood is centralized in deeper tissue layers^7,8,10^. In contrast, TCI is proposed to indicate blood redistribution toward the skin to facilitate muscular heat reduction^8^. According to recent research, IRT related parameter such as T_sk_ and TCI are considered to capture those vasomotor adjustments rather than changes in evaporation^6,8^, as suggested by earlier studies analyzing skin temperatures^34,35^. This implies that asymmetrical distribution of sweat glands over the body is also of lesser concern. Derived from PP_sr_ calculations^8^, TCI is assumed to represent perforator vessels extending perpendicularly from muscle and adipose tissue to the subdermal and dermal plexus^36^. These vessels appear as a tree-like pattern in thermographic images with rising core temperatures^10^. Functionally, perforator vessels promote convective and conductive heat transfer by redirecting blood toward the skin surface^8^. During exercise, it was shown that TCI is sensitive to exercise intensity and correlates more strongly with selected internal load parameters compared to T_sk_^5^.

Adding to the literature, we show that TCI reliability is dependent on (i) exercise intensity and (ii) muscle groups and (iii) the exact physiological response evoked by the same exercise stimulus between two same testing conditions. Our results demonstrated a tendency toward good reliability at higher intensities and in ROIs that contribute more to exercise performance, whereas reliability of TCI was poor at lower intensities and in ROIs that are less active during exercise. We can only speculate on the reasons why TCI reliability increases with increasing intensity. However, given that thermal skin patterns are a consequence of increasing muscle temperatures^8^, rest or low intensity exercises might not sufficiently induce the redirection of blood to the skin leading to higher inconsistencies in TCI between test days. When exercise intensity increases, elevated energy metabolism results in rising heat which needs to be dissipated to maintain performance and to reduce the likelihood of heat stress^2^. Consequently, perforasomes are getting more activated^5,8^ and can be potentially assessed using the TCI^5^ with increasing reliability. In line with this consideration, previous work reported a shift in core temperature threshold leading to a delay in cutaneous vasodilation during dynamic exercise compared to rest^2,13^. This shifted cutaneous vasodilation may have contributed to the poor reliability observed at lower intensities. Adding to this, the calculation of the TCI reflects the subtraction of extreme high and low temperature values in a selected ROI aiming to capture fine biological structures. Potentially, future studies should examine more precise image analysis methods based on pattern recognition to ensure higher validity and reliability of thermal skin patterns identification. Another reason for compromised TCI reliability during rest or lower intensities might be in the calculations of the reliability parameters itself^30^. The limited scale range of TCI may inflate CV values due to low mean values. Therefore, CV values should be interpreted with caution and are reported in the respective tables for completeness but are not discussed further. Our findings indicate improved reliability of TCI in the anterior thigh compared to the posterior thigh during increased external load. One possible explanation is that the anterior thigh constitutes the primary power-producing musculature during cycling^37^. Since thermal pattern activation reflects heat dissipation from deep muscle layers with increasing intensity^8,10^, our results suggest that TCI yields more reliable measurements in muscles most relevant to exercise performance.

Even though the same external load was applied under same laboratory conditions, our results show that heart rate (β = -3.53, *p* < 0.05) differed between the two tests. Although our results should be interpreted cautiously due to the assessment of multiple physiological parameters, the significant difference in heart rate aligns with outcomes of previous research. Zinner et al. examined three incremental exercise tests per week for nine weeks of twelve participants^38^. The results showed a mean day-to-day variability in response to incremental exercise tests for heart rate of 1.1–2.1%^38^ indicating small but noticeable differences even in standardized conditions. Our results suggest that these differences could potentially affect TCI (Influence of HR on TCI: β = 0.46, *p* < 0.001) and consequently its reliability. In fact, our findings indicated that TCI is responsive to physiological changes, with over 40% of the variance across the two tests being explained by the selected physiological responses (marginal R^2^: 0.412). Moreover, TCI exhibited greater within-subject variability than between-subject variability (σ² = 0.48 > τ₀₀ = 0.17), further supporting its responsiveness to physiological changes. Our findings suggest that the lower reliability of TCI may partly result from its responsiveness to physiological fluctuations, suggesting a reliability–responsiveness trade-off rather than solely reduced measurement precision. Future dose–response studies should investigate the relationships between established internal load parameters (e.g., heart rate, blood lactate, and V̇O₂) and IRT-derived measures to quantify the relative contributions of biological variability and measurement error to TCI and T_sk_. Future investigation should further analyze the reliability of the TCI and internal load relationship to provide more precise results on the physiological changes. Additionally, day-to-day variability of physiological parameters and the sensitively responsiveness of TCI suggest that future research should investigate how many repeated measurements are required to establish a robust and stable baseline of TCI values. Similar approaches have been applied in studies on heart rate variability, also characterized by high day to day variability, demonstrating that averaging HRV values over seven data points provides superior methodological validity compared to relying on a single measurement^39,40^. An additional study analyzed that averaging a minimum of at least three data points of HRV are required to provide valid outcomes^41^.

Overall, TCI appears to sensitively reflect aspects of internal load, but this can also result in compromised reliability between tests, even if the external load is the same. Future studies should further investigate TCI sensitivity in various environmental conditions such as high ambient temperatures or high humid conditions.

### Reliability of T_sk_ and its responsiveness to physiological parameters following two incremental cycling tests

In response to high-intensity endurance exercises, T_sk_ declines and reflects the vasoconstriction processes of peripheral arterioles to redistribute blood to the activated muscles to meet oxygen demands^6,7^. Previous literature analyzing the reliability of T_sk_ showed in 14 male recreational athletes who performing two incremental running tests that T_sk_ has an ICC of 0.52 and a CV of 1.58% at rest and an ICC of 0.56 and a CV of 1.50% dring exercise^26^. These findings are in contrast with our results showing good to excellent ICC values (0.74–0.94, except for one occasion at increment Pre) and lower CVs (0.70–1.48). Several methodological factors may explain the discrepancies. First, the previous study relied exclusively on the spot analysis function of the FLIR E40bx camera^26^. Spot analysis captures temperature from only a small pixel cluster, producing a point measurement rather than a broader skin assessment. Since skin thermal patterns are highly individual and spatially heterogeneous, this method risks measuring a local hot or cold spot, yielding non-representative values^8^. This limitation may have been amplified by the hot exercise environment in the study by James et al.^26^. In comparison with the moderate temperatures in our study, the hot environment could additionally cause a changed core-to-skin temperature gradient, leading to increased skin blood flow^2^. A further methodological difference is the spatial resolution of the used thermal camera: The FLIR E40bx (160 × 120 pixels) was used by James et al.^26^, whereas we employed the FLIR E54 (320 × 240 pixels), enabling more detailed thermal mapping. Finally, the prior study calculated overall T_sk_ using measurements from the chest, arm, thigh, and calf. However, research has shown that different ROIs display distinct post-exercise T_sk_ responses^8,42^, which may further explain the differences between our and the previous reported findings^26^. Given the methodological deficiencies in previous studies, our findings advance the current understanding of T_sk_ by demonstrating its good to excellent reliability across two incremental tests.

Additionally, our results demonstrated a significant increase in T_sk_ from Day 1 to Day 2 (β = 0.10 SD, *p* = 0.001) and corresponding raw change was 0.12 °C (95% CI: 0.05; 0.20 °C), which might contradict the previously reported high reliability of T_sk_. However, this difference is well below the SEM_A,1_ (0.28 °C to 0.53 °C) and MDC95_A,1_ (0.78 °C to 1.47 °C), indicating that the increase in T_sk_ is within the error range and therefor its meaningfulness is reduced. The effect likely reflects a small systematic bias between test days rather than a physiologically relevant change detectable in single participants. Although our results demonstrated good to excellent reliability for T_sk_ during incremental exercise tests, future investigations should interpret outcomes that are smaller than the reported SEM (0.28 °C to 0.53 °C) and MDC95 values (0.78 °C to 1.47 °C) with caution.

While our results demonstrated significant influences of lactate (β = -0.11, *p* < 0.001), heart rate (β = 0.26, *p* < 0.001), V̇O_2_ (β = -0.21, *p* = 0.010), energy expenditure (β = -0.08, *p* = 0.005), and RPE (β = -0.29, *p* < 0.001) on T_sk_, lactate and heart rate indicated inconsistent influences across the two tests (Test2:Lactate, β = 0.11, *p* = 0.010; Test2:Heart rate, β = -0.22, *p* = 0.001), reflecting a limited responsiveness of T_sk_ to these physiological parameters. Given that multiple physiological parameters were assessed, this finding should be interpreted cautiously. However, our findings reinforced the limited association between T_sk_ and physiological responses, showing that only a small part of the total variance in T_sk_ across both tests was explained by the selected physiological parameters (marginal R² = 0.104). In contrast, a large proportion of the total variance across both tests was attributable to subject-specific characteristics (τ₀₀ = 0.67 > σ² = 0.28; conditional R² = 0.735) potentially including individual thermoregulatory and morphological attributes (e.g., tissue composition, vascular regulation, and chronic training adaptations) which appear to exert a stronger influence on T_sk_ than acute physiological load. While previous studies reported correlations between T_sk_ and physiological parameters such as heart rate, lactate, V̇O_2_, energy expenditure, and RPE^4,5,13,43,44^, our results depict a more nuanced picture, indicating that the combined influence of multiple physiological parameters across repeated measurements, as encountered in real-world settings, is less consistent on T_sk_.

In conclusion, our findings suggest that while T_sk_ is primarily determined by subject-specific characteristics, physiological responses exert a smaller and less consistent influence on T_sk_.

### Strength, limitations and future research

A strength of our study was the concomitant analysis TCI and T_sk_ regarding their reliability and the effect of selected cardiovascular, metabolic and subjective parameters across two incremental cycling tests. While we show the reliability of acute responses of TCI and T_sk_ during two cycling occasions, future studies should evaluate adaptations of TCI and T_sk_ following different training programs (e.g. high intensity interval training or high-volume training) and to reveal if there are preferable training programs to develop training programs for optimized thermoregulation which can be detected by the TCI.

To enable a holistic reliability analysis, future studies should consider approaches to clearly distinguish between measurement error and biological variability, as the present test-retest design does not permit the differentiation between the two components. Although research indicated that TCI is suitable for detecting thermal patterns using infrared thermography, and our findings demonstrated improved reliability of TCI in muscles most contributing to the exercise performance at higher intensities, there remains a lack of studies assessing whether TCI is related to vasodilated perforasomes and a redistribution of blood from deep muscle layers to the skin surface. Future research should examine TCI in conjunction with blood flow (e.g. via laser-Doppler flowmetry) and muscle temperature measures in response to increasing exercise intensity to elucidate whether TCI validly represents activated perforasomes. In this context, the shift of warm blood pooling in response to an increasing internal thermal signal may be associated with TCI.

A limitation of our study is that, in order to control for influencing factors, our study was performed indoors without air flow on a stationary bicycle ergometer. Future studies should assess TCI during real life conditions outdoors during training and competition. In addition, the homogeneity of the study cohort may have affected the ICC estimates by reducing between-subject variability. Therefore, the reported ICC values should be interpreted with caution and may have limited generalizability. Since no a priori sample size calculation was conducted and sample size was determined by the availability of eligible participants, small effects, particularly in the mixed-effects interaction analyses, should be interpreted with caution.

While we show that TCI can be reliable, multiple baseline assessments may be advisable to improve the stability of individual baseline values and, consequently, the reliability of subsequent measurements. Despite improved reliability of the TCI at higher intensities and in ROIs predominantly contributing to the power-production, future research should explore more nuanced image analysis methods stemming from biological or medical fields. While existing approaches such as TCI or entropy analysis rely on mathematical formulations that capture extreme values or the degree of temperature distribution, analytical techniques capable of detecting iteratively emerging spatial patterns within ROIs may enhance the validity and reliability of thermal assessments. In line with these considerations, future studies should assess TCI and T_sk_ in different populations to strengthen the generalizability of the reported results, thereby facilitating the usage of IRT related parameter in research and practice.

### Practical recommendations

When interpreting TCI, practitioners should account for the influence of exercise intensity, targeted muscle groups, and the associated physiological responses, as these factors affect the reliability of TCI measurements. Moreover, the inherent measurement errors of both TCI (Lowest error range at intensity Warm-up in anterior thigh; SEM_A,1_: 0.29 °C to 0.32 °C, MDC95_A,1_: 0.81 °C to 0.88 °C) and T_sk_ (Lowest error range at intensity 100–200 W in anterior thigh; SEM_A,1_: 0.28 °C to 0.29 °C, MDC95_A,1_: 0.78 °C to 0.79 °C) should be considered to avoid overinterpretation of small fluctuations within these error margins. To enhance interpretability and reliability, it is recommended to establish an individual baseline through multiple TCI assessments, particularly when using this parameter to evaluate pre- and post-intervention or training-related changes. This is supported by our results indicating that repeated measurements of TCI enhance reliability as reflected by improved values for ICC_A,2_ (Highest reliability at intensity 100–200 W in anterior thigh: 0.86 °C to 0.89 °C), SEM_A,2_ (Lowest error range at intensity 100–200 W in anterior thigh; 0.23 °C to 0.24 °C) and MDC95_A,2_ (Lowest error range at intensity 100–200 W in anterior thigh; 0.62 °C to 0.66 °C).

## Conclusion

Our study demonstrated a tendency that TCI reliability, measured by infrared thermography, ranged from good during high intensities and in ROIs more contributing to exercise performance to poor in lower intensities and in ROIs less active during exercising. Additionally, TCI appeared to be responsive to selected internal load parameters (most prominently heart rate), indicating its potential to reflect acute physiological changes. Therefore, TCI should be interpreted during standardized exercise testing in conjunction with these parameters. While T_sk_ showed good to excellent reliability (except for two occasions at increment Pre), our results suggest that T_sk_ is more influenced by subject-related factors than by nuanced acute physiological responses during exercise testing, suggesting its potential as a stable individual characteristic rather than a dynamically responsive indicator of internal load.

## Data Availability

Data are available from the corresponding author upon reasonable request.
